# Supplementary material for: Animal movement tools (amt): R package for managing tracking data and conducting habitat selection analyses
Source: Ecol Evol. 2019 Feb 5;9(2):880–90. doi: 10.1002/ece3.4823 (PMC6362447; doi:10.1002/ece3.4823)
Supplement: Supplementary file 1 [file ECE3-9-880-s001.pdf]

Supplement to: Movement Tools (amt): R Package for Managing Tracking Data and Conducting Habitat Selection Analyses

Johannes Signer, John Fieberg & Tal Avgar

July 16, 2018

Supplement S1

Source code to reproduce the two examples.

Example 1: One animal

```
library(raster)
library(lubridate)
library(amt)
library(tidyverse)
set.seed(123)

dat <- read_csv("data/Martes pennanti LaPoint New York.csv") %>%
  filter(!is.na(‘location-lat‘)) %>%
  select(x = ‘location-long‘, y = ‘location-lat‘,
         t = ‘timestamp‘, id = ‘tag-local-identifier‘) %>%
  filter(id %in% c(1465, 1466, 1072, 1078, 1016, 1469)) # for example 2
dat_1 <- dat %>% filter(id == 1016)


dat_1 <- amt::make_track(dat_1, x, y, t, crs = sp::CRS("+init=epsg:4326")) %>%
  amt::transform_coords(sp::CRS("+init=epsg:5070"))

summarize_sampling_rate(dat_1)

stps <- amt::track_resample(dat_1, rate = minutes(10), tolerance = minutes(1)) %>%
  filter_min_n_burst(min_n = 3) %>% steps_by_burst() %>%
  time_of_day(include.crepuscule = FALSE)

str(stps, width = 80, strict.width = "no", nchar.max = 80, give.attr = FALSE)
```

```
land_use <- raster("data/landuse_study_area.tif")
wet <- land_use == 90
names(wet) <- "wet"

eda1 <- stps %>% extract_covariates(wet, where = "start") %>%
  mutate(landuse = factor(wet, levels = c(0, 1), labels = c("other", "forested wetland")))

# Plot 1 ------------------------------------------------------------------

p1 <- eda1 %>% select(landuse, tod = tod_end_, sl_, ta_) %>%
  gather(key, val, -landuse, -tod) %>%
  filter(key == "sl_") %>%
  ggplot(., aes(val, group = tod, fill = tod)) + geom_density(alpha = 0.5) +
  facet_wrap(~ landuse, nrow = 2) +
  xlab("Step length [m]") + theme_light() +
  ylab("Density") +
  theme(legend.title = element_blank())

p2 <- eda1 %>% select(landuse, tod = tod_end_, sl_, ta_) %>%
  gather(key, val, -landuse, -tod) %>%
  filter(key == "ta_") %>%
  ggplot(., aes(val, group = tod, fill = tod)) + geom_density(alpha = 0.5) +
  facet_wrap(~ landuse, nrow = 2) +
  xlab("Turn angle") + theme_light() +
  theme(legend.title = element_blank(),
        axis.title.y = element_blank())

library(cowplot)
pg1 <- plot_grid(
  p1 + theme(legend.position = "none"),
  p2 + theme(legend.position = "none"), rel_widths = c(1, 1)
)

leg <- get_legend(p1)
plot_grid(pg1, leg, rel_widths = c(1, 0.1))

ggsave("fig_eda_1_animal.pdf", width = 20, height = 18, units = "cm")

#####################################

# fit the model
m1 <-stps %>% amt::random_steps(n = 9) %>%
  amt::extract_covariates(wet) %>%
  amt::time_of_day(include.crepuscule = FALSE) %>%
  mutate(log_sl_ = log(sl_)) -> d1

m1 <- d1 %>% amt::fit_issf(case_ ~ wet +  sl_  + wet:tod_end_+  sl_:tod_end_ + strata(step_id_))
m1 <- d1 %>% amt::fit_issf(case_ ~ wet + log_sl_ + wet:tod_end_+ log_sl_:tod_end_ + strata(step_id_))
m1 <- d1 %>% amt::fit_issf(case_ ~ wet + log_sl_ + sl_  + wet:tod_end_+ log_sl_:tod_end_ + sl_:tod_end_ + strata(s

AIC(m1$model)
summary(m1)

s <- summary(m1$model)$coefficients
```

```
s
print(xtable::xtable(s, digits = 4,
                     type = "latex",
                     caption.placement = "top"))

shape <- sl_shape(m1)
scale <- sl_scale(m1)

shape_adj_day <- amt::adjust_shape(shape, coef(m1)["log_sl_"])
shape_adj_night <- amt::adjust_shape(shape, coef(m1)["log_sl_"]) +
  coef(m1)["log_sl_:tod_end_night"]

scale_adj_day <- amt::adjust_scale(scale, coef(m1)["sl_"])
scale_adj_night <- amt::adjust_scale(scale, coef(m1)["sl_"]) +
  coef(m1)["sl_:tod_end_night"]

# speed
speed_day <- shape * scale_adj_day
speed_night <- shape * scale_adj_night

speed_day <- shape_adj_day * scale
speed_night <- shape_adj_night * scale

speed_day <- shape_adj_day * scale_adj_day
speed_night <- shape_adj_night * scale_adj_night

scale
shape

shape_adj_day
shape_adj_night

x <- seq(1, 500, 1)
plot(x, dgamma(x, shape = shape_adj_night, scale = scale_adj_night), type = "l")
lines(x, dgamma(x, shape = shape_adj_day, scale = scale_adj_day), type = "l")


# Bootstrap everything
mod_data <- stps %>% amt::random_steps(n = 9) %>%
  amt::extract_covariates(wet, where = "end") %>%
  amt::time_of_day(include.crepuscule = FALSE) %>%
  mutate(log_sl_ = log(sl_), cos_ta_ = cos(as_rad(ta_)))

strata <- unique(mod_data$step_id_)
n <- length(strata)

bt <- replicate(1000, {
  m_boot <- mod_data[mod_data$step_id_ %in% sample(strata, n, TRUE), ] %>%
    amt::fit_clogit(case_ ~ wet + log_sl_ + sl_ + wet:tod_end_ +
                      sl_:tod_end_ + log_sl_:tod_end_ + strata(step_id_))

  scale_adj_day <- amt::adjust_scale(shape, coef(m_boot)["sl_"])
  scale_adj_night <- amt::adjust_scale(shape, coef(m_boot)["sl_"]) +
    coef(m_boot)["sl_:tod_end_night"]

  shape_adj_day <- amt::adjust_shape(shape, coef(m_boot)["log_sl_"])
```

```
  shape_adj_night <- amt::adjust_shape(shape, coef(m_boot)["log_sl_"]) +
    coef(m_boot)["log_sl_:tod_end_night"]

  ## speed
  c(shape_adj_day * scale_adj_day,
    shape_adj_night * scale_adj_night)
})

bt2 <- data_frame(
  rep = 1:ncol(bt),
  day = bt[1, ],
  night = bt[2, ]
) %>% gather(key, val, -rep)


bt2 %>% group_by(key) %>% summarise(lq = quantile(val, 0.025),
                                    me = median(val),
                                    mean = mean(val),
                                    uq = quantile(val, 0.975))

# m/min
bt2 %>% group_by(key) %>% summarise(lq = quantile(val, 0.025) / 10,
                                    me = median(val) / 10,
                                    mean = mean(val) / 10,
                                    uq = quantile(val, 0.975) / 10)

## Simulate ud
wet_c <- crop(wet, amt::bbox(dat_1, spatial = TRUE, buff = 1e3))

mk <- amt::movement_kernel(scale, shape_adj_day, wet_c)
hk <- amt::habitat_kernel(list(wet = coef(m1)["wet"]), wet_c)


system.time(ssud_day <- amt::simulate_ud(
  mk, hk,
  as.numeric(stps[1, c("x1_", "y1_")]),
  n = 1e7))
plot(ssud_day)

system.time(tud_day <- amt::simulate_tud(mk, hk, as.numeric(stps[1501, c("x1_", "y1_")]), n = 72, n_rep = 5e3))
plot(tud_day)


# night
mk <- amt::movement_kernel(scale, shape_adj_night, wet_c)
hk <- amt::habitat_kernel(list(wet = coef(m1)["wet"] +
                                 coef(m1)["wet:tod_end_night"]), wet_c)

system.time(ssud_night <- amt::simulate_ud(
  mk, hk, as.numeric(stps[1, c("x1_", "y1_")]), n = 1e7))
plot(ssud_night)

system.time(tud_night <- amt::simulate_tud(mk, hk, as.numeric(stps[1501, c("x1_", "y1_")]), n = 72, n_rep = 5e3))
plot(tud_day)
plot(tud1 <- crop(tud_day, extent(c(1778000, 1782000, 2412000, 2415000))))
plot(tud2 <- crop(tud_night, extent(c(1778000, 1782000, 2412000, 2415000))))
```

```
pllog <- list(
  geom_raster(),
  coord_equal(),
  scale_fill_continuous(low = "white", high = "red",
                        tran = "log10", na.value = "white"),
  scale_y_continuous(expand = c(0, 0)),
  scale_x_continuous(expand = c(0, 0)),
  theme_light(),
  theme(legend.position = "none"))

pl <- list(
  geom_raster(),
  coord_equal(),
  scale_fill_continuous(low = "white", high = "red", na.value = "white"),
  scale_y_continuous(expand = c(0, 0)),
  scale_x_continuous(expand = c(0, 0)),
  theme_light(),
  theme(legend.position = "none"))

r1 <- data.frame(rasterToPoints(mk))
p1 <- ggplot(r1, aes(x, y, fill = d)) + pllog + ggtitle("Movement kernel (night)")

r2 <- data.frame(rasterToPoints(hk))
p2 <- ggplot(r2, aes(x, y, fill = layer)) +  pl + ggtitle("Habitat kernel (night)")


r1 <- data.frame(rasterToPoints(tud1))
p3 <- ggplot(r1, aes(x, y, fill = layer)) + pllog + ggtitle("Transient UD (day)")

r2 <- data.frame(rasterToPoints(tud2))
p4 <- ggplot(r2, aes(x, y, fill = layer)) +  pllog + ggtitle("Transient UD (night)")


r1 <- data.frame(rasterToPoints(ssud_day))
p5 <- ggplot(r1, aes(x, y, fill = layer)) + pl + ggtitle("Steady state UD (day)")

r2 <- data.frame(rasterToPoints(ssud_night))
p6 <- ggplot(r2, aes(x, y, fill = layer)) +  pl + ggtitle("Steady state UD (night)")

cowplot::plot_grid(p1, p2, p3, p5, p4, p6, ncol = 2, labels = "AUTO")



ggsave("img/fig_one_animal1.pdf", height = 20, width = 24, units = "cm")
```

Example 2: Many animals

```
library(ggplot2)
library(raster)
library(lubridate)
library(amt)
library(parallel)
```

```
dat <- read_csv("data/Martes pennanti LaPoint New York.csv") %>%
  filter(!is.na(‘location-lat‘)) %>%
  select(x = ‘location-long‘, y = ‘location-lat‘,
         t = ‘timestamp‘, id = ‘tag-local-identifier‘) %>%
  filter(id %in% c(1465, 1466, 1072, 1078, 1016, 1469))

dat_all <- dat %>% nest(-id)
dat_all$sex <- c("f", "f", "f", "m", "m", "m")
dat_all <- dat_all %>%
  mutate(trk = map(data, function(d) {
    amt::make_track(d, x, y, t, crs = sp::CRS("+init=epsg:4326")) %>%
      amt::transform_coords(sp::CRS("+init=epsg:5070"))
  }))
dat_all %>% mutate(sr = lapply(trk, summarize_sampling_rate)) %>%
  select(id, sr) %>% unnest

land_use <- raster("data/landuse_study_area.tif")
rcl <- cbind(c(11, 12, 21:24, 31, 41:43, 51:52, 71:74, 81:82, 90, 95),
             c(1, 1, 2, 3, 3, 3, 2, 5, 5, 5, 5, 5, 5, 5, 5, 5, 8, 8, 1, 1))
# water, dev open, dev, barren, forest, shrub and herb, crops, wetlands
# 1: water, wetlands
# 2: developed (open)
# 3: developed (other)
# 5: forest, herbaceouse
# 8: crops
lu <- reclassify(land_use, rcl, right = NA)
names(lu) <- "landuse"

m1 <- dat_all %>%
  mutate(steps = map(trk, function(x) {
    x %>% amt::track_resample(rate = minutes(10), tolerance = seconds(120)) %>%
      amt::filter_min_n_burst() %>%
      amt::steps_by_burst() %>% amt::random_steps() %>%
      amt::extract_covariates(lu, where = "both") %>%
      mutate(landuse_end = factor(landuse_end))
    }))


m1 <- m1 %>% mutate(fit = map(steps, ~ amt::fit_issf(., case_ ~ landuse_end +
                                                        strata(step_id_))))


d2 <- m1 %>% mutate(coef = map(fit, ~ broom::tidy(.x$model))) %>%
  select(id, sex, coef) %>% unnest %>%
  mutate(id = factor(id)) %>% group_by(term) %>%
  summarize(
    mean = mean(estimate),
    ymin = mean - 1.96 * sd(estimate),
    ymax = mean + 1.96 * sd(estimate)
  )

d2$x <- 1:nrow(d2)


p1 <- m1 %>% mutate(coef = map(fit, ~ broom::tidy(.x$model))) %>%
  select(id, sex, coef) %>% unnest %>%
```

```
  mutate(id = factor(id)) %>%
  ggplot(., aes(x = term, y = estimate, group = id, col = id, pch = sex)) +
  geom_rect(mapping = aes(xmin = x - .4, xmax = x + .4, ymin = ymin,
                          ymax = ymax), data = d2, inherit.aes = FALSE,
                  fill = "grey90") +
  geom_segment(mapping = aes(x = x - .4, xend = x + .4,
                             y = mean, yend = mean), data = d2, inherit.aes = FALSE,
                  size = 1) +
  geom_pointrange(aes(ymin = conf.low, ymax = conf.high),
                  position = position_dodge(width = 0.7), size = 0.8)  +
  geom_hline(yintercept = 0, lty = 2) +
  labs(x = "Habitat", y = "Relative Selection Strength") +
  theme_light() +
  scale_x_discrete(labels = c("Developed (open)", "Developed (other)", "Natural", "Crops"))

p1

ggsave("img/fig_all_animals.pdf", width = 24, height = 12, units = "cm")
```

Supplement S2

The likelihood of the parameters, $\boldsymbol{\beta}$, given the data, $\boldsymbol{X}$:

$$L(\boldsymbol{\beta}|\boldsymbol{X}) = \prod_{i=1}^{n} \frac{\exp(\text{wet}_i(\beta_1 + \beta_2 \text{tod}_i) + \text{log\_sl}_i(\beta_3 + \beta_4 \text{tod}_i))}{\sum_{j=0}^{R} \exp(\text{wet}_{ij}(\beta_1 + \beta_2 \text{tod}_i) + \text{log\_sl}_{ij}(\beta_3 + \beta_4 \text{tod}_i))}$$

Where $\beta_1, \beta_2, \beta_3, \beta_4$ are the parameters (the components of $\boldsymbol{\beta}$), whereas tod_i, log_sl_i are the covariates for step i (the components of $\boldsymbol{X}$). Each step i is coupled with R random steps; steps that the animal could have taken, but did not. Together with the observed step ($j = 0$), these random steps comprise the contrast set in the denominator.
